# Supplementary material for: Polymorphisms in Pfkelch13 domains before and after the introduction of artemisinin-based combination therapy in Southwest Nigeria
Source: PLoS One. 2025 Mar 31;20(3):e0316479. doi: 10.1371/journal.pone.0316479 (PMC11957316; doi:10.1371/journal.pone.0316479)
Supplement: Supporting information 2 — (ZIP) [file pone.0316479.s002.zip › 201KN1R_PREMIX_Plate_KELCH1_F08.pdf]

Samples: 13846
Bases: 825
Average spacing: 17

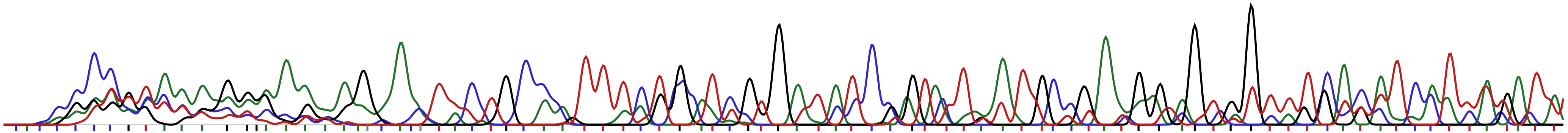

C A C C C C C G T A A A G G G A A A A A A - C A C - T A C T G C A A T T T C T G A T C G T G A T A T C C G T T T A A T T C C T A T G G A G T G G T T T C A T A T C C T T A T A T T

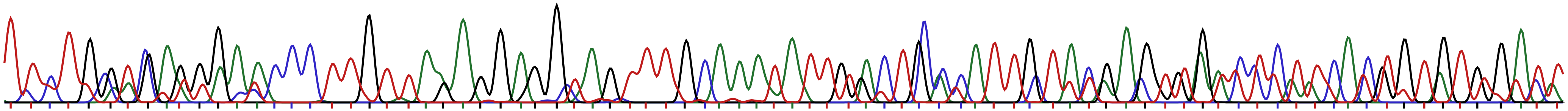

T T C T G G T G A T G G A A C C C T T G T T A G A G G A G G T A G T T T G C A A A T A T T T A C T C C C A T T C T A T G A G T T G T C T C T T C A C T G T G T G G A T T

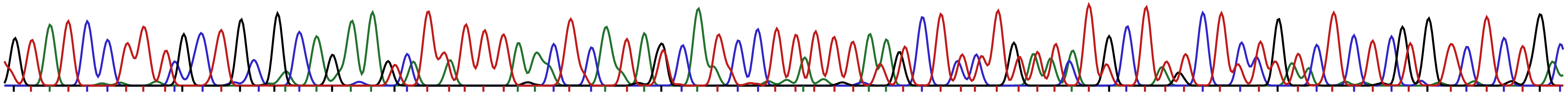

G T A T C C T T T C G C T G C G A C A G A A G C T T T T T A A C T C A T A G C A T C C T T A T T T A A T C T T T T T T T A T G C T T T C T C T G T C T C T C T G T C T C T G C

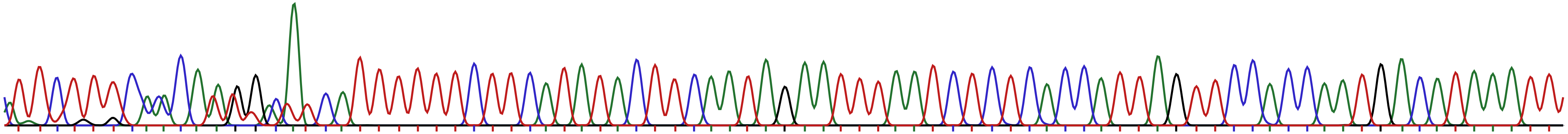

T T C T T T C A A C A A G G C A T C A T T T T T T C T T C A T A T A C T T C A A T A G A A T T T A A T C T C T C A C C A T T A G T T C C A C C A A T G A C A T A A A T T

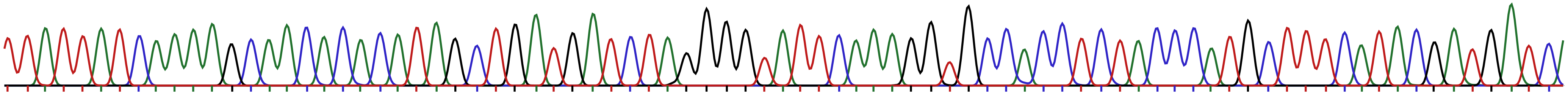

T T A T T A T C A A A A G C A A C A C A C A T A G C T G A T G A T C T A G G G G T A T T C A A A G G T G C C A C C T C T A C C C A T G C T T T C A T A C G A T G A T C

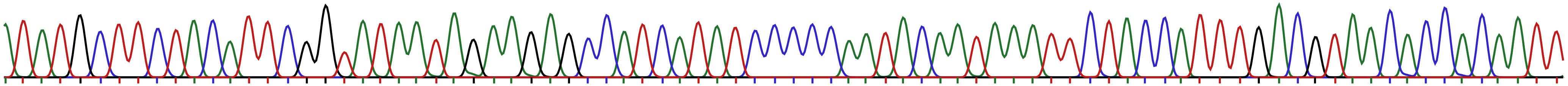

A T A T G C T T C T A C A T T C G G T A T A A T A G A A G A G C C A T C A T A T C C C C C A A T A C A A T A A A T T C T A C C A T T T G A C G T A A C A C C A C A A T T

Samples: 13846
Bases: 825
Average spacing: 17

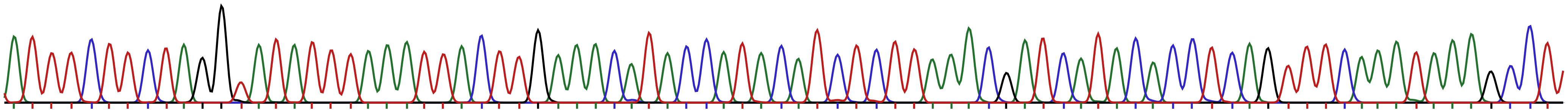

520 530 540 550 560 570 580 590

A T T T C T T C T A G G T A T A T T T A A A T T A C T T G A A A C A T A C C A T A C A T C T C T T A A A C G A T C A T A C A C C T C A G T T T C A A A T A A A G C C T

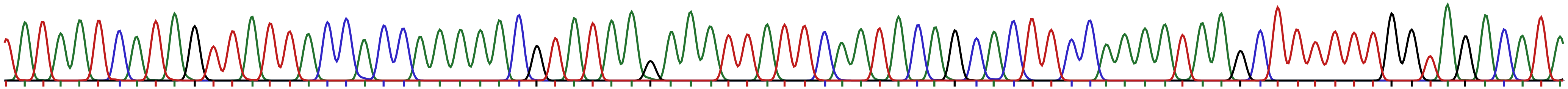

600 610 620 630 640 650 660 670

T A T A A T C A T A G T T A T T A C C A C C A A A A A C G T A T A A G A A A T T A T T C A A T A C A G C A C T T C C A A A A T A A G C T T T T T G G T A G A C A T A

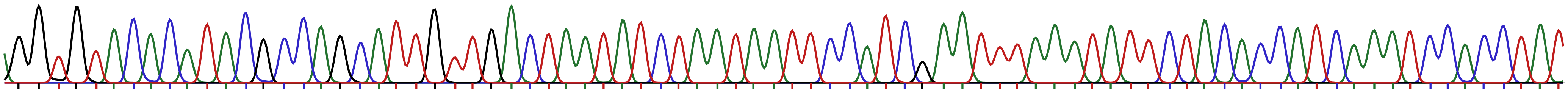

680 690 700 710 720 730 740 750 760

G G T G T A C A C A T A C G C C A G C A T T G T T G A C T A A T A T C T A A T A A T T C C A T C G A A T T T A A A T A T T C T A C A C C A T C A A T C C A C C T A T

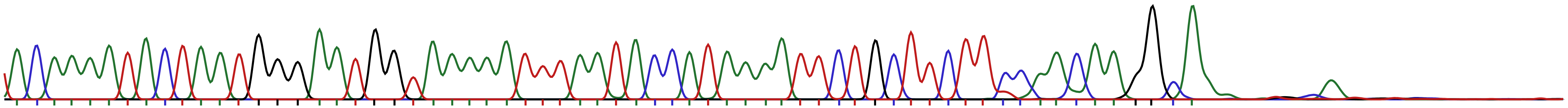

770 780 790 800 810 820

A C A A A A T A C T A A T G G G A A T G G T A A A A A T T T A A T A C C A T A A A A T T C T G C T T C T T C C A A C A A G G C A

Samples:	13846
Bases:	825
Average spacing:	17
